# Screening for Fabry Disease in Left Ventricular Hypertrophy: Documentation of a Novel Mutation

**DOI:** 10.5935/abc.20150090

**Published:** 2015-08

**Authors:** Ana Baptista, Pedro Magalhães, Sílvia Leão, Sofia Carvalho, Pedro Mateus, Ilídio Moreira

**Affiliations:** Centro Hospitalar de Trás-os-Montes e Alto Douro, Unidade de Vila Real - Portugal

**Keywords:** Fabry disease / complications, Hypertrophy, left ventricular, Alpha-Galactosidase / genetics

## Abstract

**Background:**

Fabry disease is a lysosomal storage disease caused by enzyme α-galactosidase A deficiency as a result of mutations in the GLA gene. Cardiac involvement is characterized by progressive left ventricular hypertrophy.

**Objective:**

To estimate the prevalence of Fabry disease in a population with left ventricular hypertrophy.

**Methods:**

The patients were assessed for the presence of left ventricular hypertrophy defined as a left ventricular mass index ≥ 96 g/m^2^ for women or ≥ 116 g/m^2^ for men. Severe aortic stenosis and arterial hypertension with mild left ventricular hypertrophy were exclusion criteria. All patients included were assessed for enzyme α-galactosidase A activity using dry spot testing. Genetic study was performed whenever the enzyme activity was decreased.

**Results:**

A total of 47 patients with a mean left ventricular mass index of 141.1 g/m^2^ (± 28.5; 99.2 to 228.5 g/m^2^] were included. Most of the patients were females (51.1%). Nine (19.1%) showed decreased α-galactosidase A activity, but only one positive genetic test − [GLA] c.785G>T; p.W262L (exon 5), a mutation not previously described in the literature. This clinical investigation was able to establish the association between the mutation and the clinical presentation.

**Conclusion:**

In a population of patients with left ventricular hypertrophy, we documented a Fabry disease prevalence of 2.1%. This novel case was defined in the sequence of a mutation of unknown meaning in the GLA gene with further pathogenicity study. Thus, this study permitted the definition of a novel causal mutation for Fabry disease - [GLA] c.785G>T; p.W262L (exon 5).

## Introduction

Establishing the cause of left ventricular hypertrophy (LVH) is a common challenge in clinical practice, given its high prevalence and the variety of diseases with which it may be associated. This is particularly relevant from the clinical standpoint because of the therapeutic implications regarding the different differential diagnoses.

Fabry disease (FD) is a rare X-linked disease caused by enzyme α-Galactosidase A (Gal A) deficiency as a result of GLA gene mutations. Most families present with a "private" mutation found only in that family and, thus, hundreds of causal mutations are currently known. This multiplicity of mutations may contribute to variations in the residual enzyme activity and the different clinical presentations. Enzyme Gal-A deficiency leads to a progressive tissue accumulation of glycosphingolipids, especially of globotriaosylceramide (Gb3), resulting in organ failure. The organs more frequently involved are the kidneys, heart, skin, central and autonomic nervous systems, eyes and auditory system. As regards the cardiac manifestations, Gb3 accumulation leads to LVH, whose etiology is difficult to distinguish from others using the common cardiac imaging methods, especially echocardiography. Currently, suspecting lysosomal storage diseases, namely FD, is key given the availability of enzyme replacement therapy, which brings an impact on disease progression. Thus, innumerable studies have been conducted to assess the prevalence of FD in risk populations. The objective of this study was to evaluate the prevalence of FD in a population of patients with LVH.

## Methods

The study was conducted between October 2010 and February 2011 in a hospital center in the region of Tras-os-Montes and Alto Douro, northern Portugal, after approval of the Institutional Ethics Committee. All patients with age > 18 years referred for transthoracic echocardiography (TTE) were considered eligible for the FD screening program. They were included if there was evidence of LVH, defined as a left ventricular (LV) mass / body surface area (BSA) ≥ 96 g/m^2^, for women, and ≥ 116 g/m^2^, for men. LV mass was calculated using the formula:

**Table:** 

LV mass = 0.8 x (1.04 [(LV internal diameter + posterior wall + septum)^3^ – (LV internal diameter)^3^]) + 0.6

Linear measurements during diastole obtained from two-dimensional echocardiography were used for the calculation. The exclusion criteria were presence of severe aortic stenosis and arterial hypertension when associated with mild LVH - LV mass/BSA < 109 g/m^2^ (women) and < 132 g/m^2^ (men), regardless of the hypertension stage. All patients enrolled gave written informed consent prior to undergoing clinical assessment, investigation of multiorgan involvement, and determination of enzyme Gal-A activity.

### Clinical assessment

After the inclusion criteria were defined, brief history taking focused on the investigation of symptoms suggestive of FD and multiorgan involvement, especially cardiac, was performed. Thus, the questionnaire included the assessment of history of bouts of pain in extremities; gastrointestinal transit or sweating abnormalities; history of stroke; and presence of shortness of breath/orthopnea or chest pain. Next, blood pressure (BP), height and weight measurements were taken, and angiokeratomas were investigated. Clinical assessment did not include family history for cardiovascular or renal diseases.

### Investigation of multiorgan involvement

The cardiac investigation was complemented by electrocardiogram (ECG) - assessment of rhythm, heart rate (HR), PR interval, conduction disturbances, and voltage criteria for LHV (Sokolow-Lyon criteria). For the investigation of renal involvement, BUN and serum creatinine levels were determined, with further estimate of the glomerular filtration rate (GFR) using the MDRD formula (Modification of Diet in Renal Disease). Random urine specimens were also collected to rule out albuminuria, using the microalbuminuria/urine creatinine ratio.

### Enzyme Gal-A activity determination

Screening for FD was based on the dried blood spot (DBS) test, with four blood spots placed in a filter paper and allowed to dry at room temperature. Enzyme Gal-A activity was determined in an outside laboratory (laboratory of metabolism, Hamburg University Medical Center). Values between 200 and 2000 pmol/spot*20 h were considered normal.

### Genetic screening

Whenever enzyme Gal-A activity was reduced, genetic screening was performed using 10 mL of blood collected in an EDTA tube with further GLA gene sequencing in the Center of Medical Genetics Doctor Jacinto de Magalhaes.

Data were submitted to descriptive analysis using the Statistical Package for the Social Sciences (SPSS) program, version 19.0 (SPSS Statistics IBM®), and were expressed as numbers or percentages or mean values ± standard deviation (SD). 95% confidence intervals (95% CI) were used when applicable.

## Results

During the study period, 75 patients had inclusion criteria; of these, 28 (37.3%) were excluded for showing arterial hypertension with mild LVH (21 patients), or severe aortic stenosis (7 patients). Screening for FD was then performed in 47 patients, of whom 24 were women (51.1%). The mean age of patients was 65.6 ± 14.5 years (ranging from 25 to 90 years). As regards their ventricular mass, the mean LV mass/BSA was 141.1 ± 28.5 g/m^2^ (99.2 to 228.5 g/m^2^) and the mean septal and posterior wall thickness was 15.3 ± 3.4 mm (10 to 24 mm) and 12.9 ± 2.1 mm (9 to 20 mm), respectively.

### Clinical characterization of the study population

Most of the study population assessed had a known history of arterial hypertension (n = 35; 74.5%), with a mean systolic BP recorded on the day of assessment of 144.2 ± 30.3 mmHg (96 to 216 mmHg). The summary review of the clinical history suggested the presence of bouts of pain in the extremities in 27.7% of patients, sweating abnormalities in 4.3%, and gastrointestinal transit abnormalities in 29.8%. No skin lesions suggestive of angiokeratoma were found in any of the patients. Eight patients (17.0%) had history of stroke, 66.0% presented with dyspnea, and 40.4% with chest pain.

Of the patients assessed, three (6.4%) were on a regular dialysis program, with the population showing a mean GFR of 81.7 ± 50.2 mL/min/1.73m^2^ (3.9 to 232.6 mL/min/1.73m^2^), as estimated from mean creatinine values of 1.4 ± 2.1 mg/dL (0.4 to 14.6 mg/dL). The prevalences of microalbuminuria and proteinuria were of 25.5% and 55.4%, respectively. Electrocardiographic assessment showed normal sinus rhythm in most of the patients (63.8%), with the remaining showing atrial fibrillation (19.1%) or pacemaker rhythm (17.1%). Their mean HR was 71 ± 15 bpm (45 to 110 bpm), PR interval of 169 ± 34 miliseconds (108 to 250 miliseconds), prevalence of atrioventricular and intraventricular conduction disturbance of 8.5% and 23.4%, respectively, with 40.4% of patients with criteria for LVH.

Nine patients, all females, showed reduced enzyme Gal-A activity (19.1%), and were therefore referred for genetic screening. Only one of the genetic studies was able to document a GLA gene mutation. Thus, the incidence of false positives using the enzymatic test with DBS was 88.9%.

### Description of the case showing GLA gene mutation

The single positive genetic test showed heterozygosis for [GLA] c.785G>T; p.W262L (exon 5) mutation, which had never been previously described in the literature as the cause of FD. The patient was a 46-year-old female recently diagnosed with arterial hypertension, who had been referred for TTE because of a brain stem stroke. In the clinical assessment, she had history of frequent episodes of bouts of pain in the extremities, especially in hands, and gastrointestinal transit abnormalities, but no angiokeratoma. The ECG revealed normal sinus rhythm, HR of 76 bpm and voltage criteria for LVH with an overload pattern. The echocardiogram showed moderate concentric LVH with grade-II diastolic dysfunction. Renal assessment revealed the presence of microalbuminuria with preserved renal function (creatinine level of 0.6 mg/dL).

**Figure 1:**
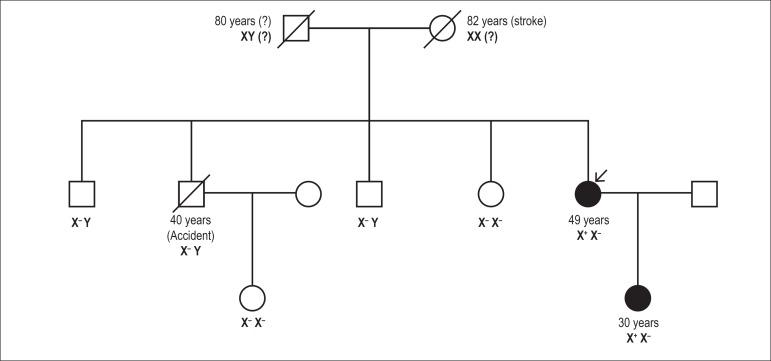
Screening for Fabry Disease in Left Ventricular Hypertrophy: Documentation of a Novel Mutation

Although the manifestations were suggestive of FD (microalbuminuria and LVH), they could also be explained by the history of hypertension and, therefore, the pathogenicity of this novel mutation had to be documented. This process involved three key steps: genetic information, demonstration of accumulation of Gb3 deposits, and family screening. Regarding the study of the mutation, the bioinformatics tools showed that it was causal, and the hypothesis of polymorphism was ruled out when it was not found in the study of 100 individuals from the population. Another aspect corroborating causality was the fact that other causal mutations had already been described in its proximity^1,2^. Tissue accumulation of Gb3 was demonstrated by cutaneous biopsy, which showed rare lysosomal inclusions with characteristics typical of Gb3 deposits in smooth muscle fibers and increased blood concentrations of lysoGb3. As regards the family study (Figure 1), it was negative for all her siblings, but positive for her daughter, who, at the age of 30 years, also presented with manifestations suggestive of FD: cornea verticilatta, peripheral neuropathic pain, and slightly increased proteins in the 24-hour urine, thus confirming the association of the mutation with the manifestations suggestive of FD. The investigation also included ruling out the involvement of other organs not defined by the study algorithm (dermatological, ophthalmological, ENT and pulmonary assessments were normal) and additional characterization by MRI of cardiac involvement, which demonstrated LVH with no late enhancement, and brain involvement, which demonstrated white matter damage. Blood pressure was also documented by ambulatory BP monitoring under medication. Thus, in a study on FD screening in patients with LVH, one case of FD was documented in association with a causal mutation - [GLA] c.785G>T; p.W262L (exon 5), which had not been previously described in the literature and is associated with neurological, cardiac and renal involvement. The Organizing Committee of Treatment of Lysosomal Storage Disorders approved enzyme replacement therapy for the patient.

## Discussion

In a population of patients with LVH, after exclusion of cases with severe aortic stenosis and arterial hypertension associated with mild LVH, we documented a FD prevalence of 2.1% (95%CI: 0.1-11.3%). This prevalence is similar to that found in other screening studies in high-risk populations (populations selected with LVH, stroke and patients undergoing dialysis)^3^, which reported prevalence rates much higher than that estimated in the general population of 0.02 to 0.09 per 10 thousand individuals^4^. However, the prevalence found should be carefully interpreted taking into consideration the small number of patients analyzed in this study. The importance of screening studies lies on two major factors. First, they seek to increase awareness on FD in target groups, in which there is a higher probability of detecting cases, and thus contribute for the definition of disease prevalence. Second, these studies also provide the opportunity to detect the disease at earlier stages, at which enzyme replacement therapy is more effective.

The available studies on the prevalence of FD in populations with LVH used different criteria for patient inclusion, whether because of different forms of assessing LVH, or because of the cutoff values defined. Most of the studies used the maximum ventricular wall thickness threshold of 13 or 15mm^4^ as an inclusion criterion, unlike in the present study, in which patient selection was based on the ventricular mass index. Although the typical manifestation of the cardiac involvement in FD is concentric LVH, several cases of asymmetrical LVH have already been documented^5^. To corroborate this fact, there are studies demonstrating a FD incidence of approximately 1.0% in populations diagnosed with hypertrophic cardiomyopathy^6^, in which asymmetrical LVH is the most frequent form of presentation. Thus, in the present study, patients with possible FD may have been excluded because increased thickness of a single ventricular wall - usually the ventricular septum, may be not associated with increased LV mass. However, this variable -left ventricular mass index, is one of the main criteria for monitoring the effectiveness of enzyme replacement therapy in the reduction of LVH^7^, and this is why it was used as a criterion for patient selection.

In this study, enzyme Gal-A activity as assessed by DBS was the method used for FD screening, whose result may be normal in up to 40% of women with FD^8^. Interpretation of the enzymatic assay is more complicated in females, because the enzyme activity may be normal or at borderline lower limits because of the phenomenon of X-chromosome inactivation (i.e., the permanent epigenetical silencing of one X chromosome creating cellular mosaicism, which explains the disease presentation in women - of delayed onset and with a higher probability of a single-organ involvement)^9^. This leads to a study limitation: the possibility of underestimating FD prevalence because of false negatives among women although, interestingly, the only positive result occurred in a female patient. The use of the DBS test was based on the fact that it is an easily-accessible screening method in the clinical practice and has advantages over enzyme activity assessment in leukocytes or fibroblasts. First, it requires only a few blood spots for dose determination and, second, it permits easy and quick specimen shipping to specialized laboratories, an important aspect in our study center, where there is no laboratory dedicated to lysosomal storage diseases. Thus, we were interested in analyzing the DBS behavior to determine its further inclusion in the assessment of female patients with suspected FD. In addition to the known false negatives - a reality for which clinicians are aware of, given documentation from multiple studies, as previously mentioned, this screening showed another limitation of the DBS test: a high incidence of false positives, which has been infrequently reported in studies^10^. Only one in nine of the patients with reduced enzyme activity (all females) had FD confirmed by genetic testing, thus resulting in 88.9% of false positives. Therefore, in the current scenario in which costs are weighted in clinical investigation, this study questions the usefulness of DBS in female patients with suspected FD (high rate of false negatives and false positives).

We observed a low incidence of unspecific manifestations, namely sweating disorders; however, gastrointestinal abnormalities and bouts of pain had an incidence of approximately 30%. This is probably explained by the fact that these are common manifestations of more prevalent diseases such as musculoskeletal and digestive disorders. On the other hand, manifestations of possible multiorgan involvement such as albuminuria had a high incidence, since they are also possible manifestations of hypertensive disease - a common finding in the study population. The assessment of these clinical and laboratory factors, which are common manifestations of FD, is a distinguishing feature of this study. Its inclusion in the screening study, unlike the usual genetic study alone in risk populations, was motivated by the interest in the definition of associations of factors with a higher probability of FD. Thus, the study results could be applied to the clinical practice, such as in the integration of findings in algorithms of etiologic studies on LVH. This way, we could limit genetic studies to certain groups selected according to criteria corroborated by a clinical trial. However, this was not possible because the sample size and the low incidence of FD limited the study power to draw conclusions on the improvement of pre-test probability. These findings were ultimately useful in the assessment of the significance of *de novo* mutations, as explained bellow.

**Figure 2:**
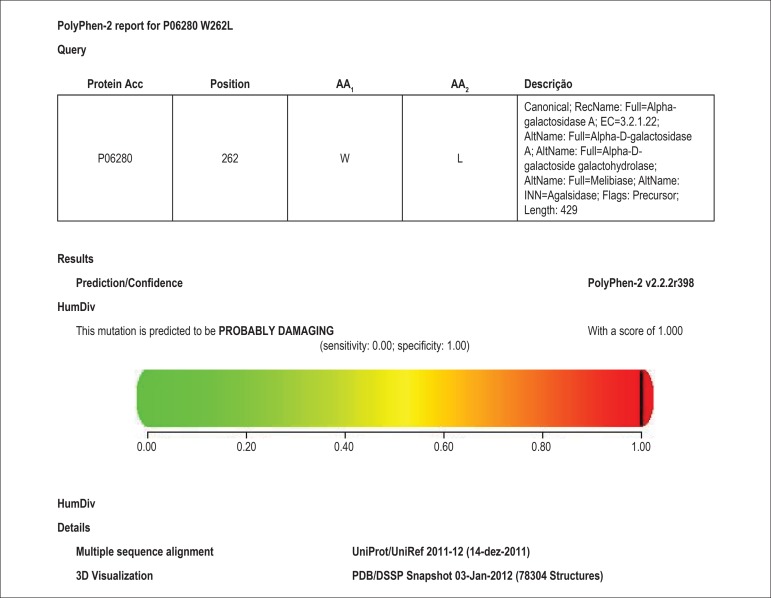
Use of bioinformatics tools to estimate the causality of the mutation.

To date, more than 600 GLA gene variants have been described and most of them is unique for each family. However, as more studies on FD screening started to be conducted, an unexpected high number of individuals with mutations of unknown significance was observed, with a prevalence rate estimated at 0.6% in high-risk populations, although only 0.12% have typical clinical manifestations or decreased enzyme activity^11^. Thus, the significance of these mutations is not easy to define, because many of the screening studies involve high-risk populations that express one single specific symptom - the inclusion criteria, LVH in the case of this study and, thus, the symptom may be not related to the GLA gene variant detected or may be an FD manifestation in its non-classic form. In this study on FD screening in a predominantly hypertensive population (74.5%) with LVH, this became a clear issue. We found a GLA gene mutation not previously described in the literature - [GLA] c.785G>T; p.W262L (exon 5), whose significance could not be defined based on the finding of LVH on TTE. The patient had been diagnosed with hypertension and, thus, the LVH findings along with the presence of microalbuminuria and history of stroke could be explained either by FD or by arterial hypertension with target-organ involvement, given that the manifestations can overlap in both situations. This led to a clinical investigation focused on proving the pathogenicity associated with the mutation, which involved three key steps: bioinformatics techniques, histological diagnosis, and family screening. The assessment of the occasional pathogenicity of the mutation involved the documentation of its absence in the study of 100 chromosomes of individuals from the general population, thus excluding the hypothesis of polymorphism. Then, we proceeded to a literature review in the search for mutations described in its proximity, which also increases the probability of pathogenicity, and found the p. W262C (c.786G>C) mutation documented by Schafer et al.^12^, and the p. W262X (c.785G>A) mutation described by Shabbeer et al.^13^. The use of bioinformatics tools defined the mutation as causal. We used the PolyPhen-2® (Polymorphism Phenotyping v2) program, which predicts the possible impact of the replacement of one amino acid on the structure and function of human proteins (Figure 2). Accumulation of Gb3 deposits - histological diagnosis of FD, was demonstrated by cutaneous biopsy, which showed sparse lysosomal inclusions with characteristics typical of Gb3 deposits exclusively in smooth muscle fibers thus corroborating the relationship between genotype and phenotype. The process was concluded with family screening focused on confirming the association of the mutation with clinical manifestations in multiple patients. Documentation of the mutation in a normotensive first-degree relative (daughter) associated with early manifestations of FD (cornea verticillata, peripheral neuropathic pain, and increased 24-hour urine proteins) also helped to establish a causal relationship between the mutation found - [GLA] c.785G>T; p.W262L (exon 5), and FD.

## Conclusions

In a population of patients with left ventricular hypertrophy, after exclusion of severe aortic stenosis and arterial hypertension associated with mild left ventricular hypertrophy, we documented a Fabry disease prevalence of 2.1%. This stresses the importance of including this disease among the differential diagnoses of left ventricular hypertrophy. This screening study also documented an issue regarding these research methods that is not related to Fabry disease - the occurrence of a mutation of unknown significance in GLA gene, and showed the clinical management required to define the role of the mutation on the development of the clinical presentation. Thus, this study allowed the definition of a novel causal mutation for Fabry disease - [GLA] c.785G>T; p.W262L (exon 5).
